# Unravelling the process of petroleum hydrocarbon biodegradation in different filter materials of constructed wetlands by stable isotope fractionation and labelling studies

**DOI:** 10.1007/s10532-021-09942-1

**Published:** 2021-04-16

**Authors:** Andrea Watzinger, Melanie Hager, Thomas Reichenauer, Gerhard Soja, Paul Kinner

**Affiliations:** 1 Institute of Soil Research, Department of Forest- and Soil Sciences, University of Natural Resources and Life Sciences, Konrad Lorenz-Strasse 24, 3430 Tulln, Austria; 2 Environmental Resources & Technologies, Energy Department, AIT – Austrian Institute of Technology GmbH, Konrad Lorenz-Strasse 24, 3430 Tulln, Austria; 3 Bioresources, Center of Health & Bioresources, AIT – Austrian Institute of Technology GmbH, Konrad Lorenz-Strasse 24, 3430 Tulln, Austria; 4 Institute for Chemical and Energy Engineering, Department of Material Sciences and Process Engineering, University of Natural Resources and Life Sciences, Muthgasse 107, 1190 Vienna, Austria

**Keywords:** Hexadecane, Decane, Biochar, Expanded clay, Phospholipid fatty acid, Stable isotope fractionation

## Abstract

Maintaining and supporting complete biodegradation during remediation of petroleum hydrocarbon contaminated groundwater in constructed wetlands is vital for the final destruction and removal of contaminants. We aimed to compare and gain insight into biodegradation and explore possible limitations in different filter materials (sand, sand amended with biochar, expanded clay). These filters were collected from constructed wetlands after two years of operation and batch experiments were conducted using two stable isotope techniques; (i) carbon isotope labelling of hexadecane and (ii) hydrogen isotope fractionation of decane. Both hydrocarbon compounds hexadecane and decane were biodegraded. The mineralization rate of hexadecane was higher in the sandy filter material (3.6 µg CO_2_ g^−1^ day^−1^) than in the expanded clay (1.0 µg CO_2_ g^−1^ day^−1^). The microbial community of the constructed wetland microcosms was dominated by Gram negative bacteria and fungi and was specific for the different filter materials while hexadecane was primarily anabolized by bacteria. Adsorption / desorption of petroleum hydrocarbons in expanded clay was observed, which might not hinder but delay biodegradation. Very few cases of hydrogen isotope fractionation were recorded in expanded clay and sand & biochar filters during decane biodegradation. In sand filters, decane was biodegraded more slowly and hydrogen isotope fractionation was visible. Still, the range of observed apparent kinetic hydrogen isotope effects (AKIE_H_ = 1.072–1.500) and apparent decane biodegradation rates (k = − 0.017 to − 0.067 day^−1^) of the sand filter were low. To conclude, low biodegradation rates, small hydrogen isotope fractionation, zero order mineralization kinetics and lack of microbial biomass growth indicated that mass transfer controlled biodegradation.

**Supplementary Information:**

The online version contains supplementary material available at 10.1007/s10532-021-09942-1.

## Introduction

The biodegradation of petroleum hydrocarbons was found as early as in 1913, when a crude oil slick on surface waters was investigated for the presence of microorganism (Söhngen 1913). Since then it became clear that a vast amount of different microorganisms can biodegrade hydrocarbons under various environmental conditions (Atlas 1981; Abbasian et al. 2015a); an ability which might have been spread by horizontal gene transfer (Rojo 2009; Abbasian et al. 2015b). In petroleum hydrocarbon contaminated environments the number of degrading bacteria increases remarkably and even species which solely rely on hydrocarbons as a carbon source evolve (Rojo 2009; Varjani 2017; Prenafeta-Boldú et al. 2019). While crude oil consists of a mixture of different aliphatic and aromatic molecules, the diesel fraction contains mainly straight chain saturated hydrocarbons with carbon atoms ranging from decane C_10_ to eicosane C_20_ and the major constituent being hexadecane C_16_. These alkanes can be biodegraded both aerobically and anaerobically. Aerobic degradation relies on the terminal or subterminal oxidation of the hydrocarbon to produce a fatty acid alcohol which is further oxidized to an aldehyde. Anaerobic degradation pathways of hydrocarbons are more diverse and can be divided in 5 groups (Abbasian et al. 2015a). The downstream enzymes necessary for complete degradation of hydrocarbon might be common for different hydrocarbons (Abbasian et al. 2015b) and among different microorganisms (Rojo 2009), and will finally lead to the production of pyruvate and CO_2_.

The biodegradation rate of a hydrocarbons is usually controlled by its bioavailability, by the transport dynamics into the cells and the first enzymatic step of oxidation. Due to the hydrophobic nature of hydrocarbons, the dissolution in water is low (Mackay et al. 2006) and microorganisms will face them as drops in the water and / or adsorbed to soil and sediments. Groundwater temperatures in central Europe [10–12 °C (Tissen et al. 2019)] will also result in hydrocarbons larger than C_15_ being present in the solid stage, which will further reduce bioavailability. To overcome the bioavailability issues, some degrading microorganisms are known to produce biosurfactants. This feat allows them to attach to the hydrocarbon droplets, to glide towards hydrocarbon sources and to create micelles which facilitate the transport in water and through the cell biomembranes into the cell (Rojo 2009). The degradation is temperature-dependent because the viscosity of hydrocarbons increases with decreasing temperature, and hence the bioavailability decreases. Also the enzymatic reaction is temperature-dependent with a Q10 of 3–4 (Atlas 1981). If hydrocarbons are present at high concentration, oxygen and nutrients limitation may impair degradation. When increase of microbial biomass can be excluded, the degradation usually follows first order kinetics. However, if degradation is controlled by transport then the overall kinetics shifts from first- to zero order.

Wetlands have been used as a recipient of various wastewaters in the past. After their purification ability was recognized, engineered wetlands—also referred to as constructed wetlands (CW)—have been designed and built since the early 1970s to primarily treat domestic wastewaters and recently also to treat industrial wastewater and contaminated groundwater (Knight 1999; Haberl et al. 2003; Imfeld et al. 2009). Examples exist for the treatment of diesel hydrocarbons in horizontal flow CWs (Salmon et al. 1998; Omari et al. 2003; Al-Baldawi et al. 2013) and vertical flow systems (Bedessem et al. 2007; Mustapha et al. 2018b, a). The removal efficiencies of diesel hydrocarbons were between 60 and 95% and benefitted from aeration, planting and interaction with the substrate. Influent concentrations and hydraulic loading will give the frame for sizing the CWs (Knight 1999). Especially if high organic loads are applied, intermittent loading of vertical CWs might increase oxygen content and consequently biodegradation.

Besides biodegradation (60%), volatilization (25%) and adsorption (5%) are notable removal process in CWs for treating petroleum hydrocarbons containing quartz sand as filter material (Salmon et al. 1998). Sorption will reduce the amount of hydrocarbons in the soil water. Consequently, less hydrocarbons are available for biodegradation and evaporation if the sorption process is much faster. By choosing a filter material which can retain contaminants after loading and slowly releases them if the pore water concentrations decrease, a sustainable system buffering peak concentrations and / or unfavorable conditions for biodegradation (e.g. cold seasons) might be established. Sands are a common filter material but expanded clay was also successfully used for the treatment of persistent organic contaminants, such as polycyclic aromatic hydrocarbons, pesticides and pharmaceuticals (Dordio et al. 2007; Nkansah et al. 2012; Dordio and Carvalho 2013) in CWs due to its high sorption capacity. Expanded clay possesses a highly interconnected macro- to mesosize pore structure. Adsorption likely occurs through hydrophobic attraction on the hydrophobic side of expanded clay surface followed by diffusion into the pores (Dordio and Carvalho 2013; Pouramini et al. 2019). Following a fast initial adsorption (6–21 h) biodegradation was enabled (Dordio et al. 2010; Nkansah et al. 2012). Besides expanded clay, biochar was recently used for the remediation of organic contaminants and proved successful especially for aliphatic hydrocarbons and in the long term (several weeks) (Bushnaf et al. 2011, 2017). Biochar retarded biodegradation in the short term (few days) and for aromatic hydrocarbons; apparently the microbial community has to adapt to the lower contaminant concentration in the pore water (Meynet et al. 2012, 2014). However, in the long term and if microorganisms are no longer substrate (hydrocarbon) limited, but rather nutrient limited, then the biodegradation rates likely approach those without biochar. Additionally, biochar can be used as a nutrient carrier to improve plant and microbial growth. Improved plant growth is beneficial for the removal of contaminants via plant associated processes such as phytodegradation, rhizodegradation, phytovolatilisation and phytoaccumulation (Imfeld et al. 2009), e.g. Mustapha et al. (2018b) recorded a threefold increase of TPH removal under vegetation.

## Materials and methods

### Constructed wetland setting

At a hydrocarbon contaminated field site in Austria, a pilot scale groundwater treatment plant consisting of four constructed wetlands (CW) with a size of 3 m × 2 m and a depth of 1.5 m has been built in summer 2012. Operation started on the 20th of September 2012. The four CWs were intermittently loaded with hydrocarbon contaminated groundwater at loading rates between 60 and 850 L m^−2^ day^−1^ (mm day^−1^) added 2–6 times a day (Supplementary material, Table 1). From July 2013 onwards, the groundwater was additionally spiked with diesel to increase total petroleum hydrocarbon (TPH) loads. The CWs were filled with three different filter materials, washed quartz sand 0–4 mm, quartz sand mixed with 3% (w/w) biochar, and expanded clay (liapor HD 1–4 mm) and all filters were planted with willow (*Salix viminalis *L.). Filter material characteristics of expanded clay and biochar are summarized in the supplementary material (Supplementary material, Tables 2 and 3). The different treatments were named according to their filter material and the suffix + to indicate an initially higher hydraulic load; (i) sand + (ii) sand (iii) expanded clay and (iv) sand and biochar. We had already shown that diesel removal efficiency was high in all filter materials (Watzinger et al. 2016). One important removal mechanisms might be biodegradation (Salmon et al. 1998; Groudeva et al. 2001). To test this, we collected samples from the pilot scale CWs and applied two stable isotope approaches to observe the fate and behavior of hydrocarbons in a batch experiment. An overview of the experimental design of both isotope approaches are given in Table 1 and described in detail below.

**Table 1:** Design of the stable isotope microcosms experiments. The number of replicates of biological (bio) and sterile microcosms, the amount of hydrocarbon applied and the monitored parameters are listed

Hexadecane (C_16_) carbon isotope labelling experiment								
CW sampling date		15.1.2014						
Subsamples		5						
Initial parameters		TPH, PLFAs, water content						

	bio	sterile	C_16_ (µL)	Monitored parameters				
Sand +	4	2	50	CO_2_	^13^C CO_2_	PLFAs	^13^C PLFAs	TPH
Sand	4	2	50	CO_2_	^13^C CO_2_	PLFAs	^13^C PLFAs	TPH
Expanded clay	4	2	50	CO_2_	^13^C CO_2_	PLFAs	^13^C PLFAs	TPH
Sand & biochar	4	2	50	CO_2_	^13^C CO_2_	PLFAs	^13^C PLFAs	TPH

Decane (C_10_) hydrogen isotope fractionation experiment								
CW sampling date		18.9.2014						
Subsamples		5						
Initial parameters		TPH, water content						

	bio	sterile	C_10_ (µL)	Monitored parameters				
Sand	5	2	40	C_10_	^2^H C_10_			
Expanded clay	5	2	15–40	C_10_	^2^H C_10_			
Sand & biochar	4	2	15–40	C_10_	^2^H C_10_			

PLFAs = Phospholipid fatty acids, TPH = total petroelum hydrocarbons

### Design of the hexadecane carbon isotope labelling experiment

In the first approach, labelled (^13^C) hexadecane was added and the mineralization and uptake into different microbial groups was monitored by ^13^C CO_2_ and ^13^C phospholipid fatty acid (PLFA) analysis. Hexadecane was chosen as it is the major compound of diesel, which was treated in the constructed wetlands. Filter material samples for microcosms experiments were collected from the CWs on the 15th of January 2014, after 482 days of operation. At this time point the CWs had received around 123,000 mm of groundwater and 60 g of TPH (Supplementary material, Table 4). For sampling, the gravel was removed from the surface and filter material (five subsamples per CWs) from 0 to 10 cm were collected along a X-shape. For the incubation experiments subsamples were mixed to give a representative sample. Both, the subsamples and mixed ones were analyzed for their TPH content and their PLFA pattern. On the same day of filter material sampling, the groundwater used to water the wetlands was also collected. Hydrochemical characteristics including EC, pH, dissolved O_2_ content and total petroleum hydrocarbon content were analyzed (Supplementary material, Table 5). The samples were transported to the lab in a cool box and 50 g of the fresh filter material was weighed into 250 mL bottles, filled with the collected groundwater until saturation and closed with a mininert cap. Four replicate microcosms were set up per treatment. Two additional microcosms per filter material were autoclaved at 121 °C, 2 bar for 20 min, and treated as sterile controls. The water content of the filter materials before incubation was 4.3, 4.9, 32.6 and 7.8 g per 100 g dry filter material for sand + , sand, expanded clay and sand & biochar, respectively. 50 µL of n-hexadecane with a δ^13^C value of 98.8 ‰ were injected into the microcosms after 2 weeks of pre-incubation. Shortly before the addition of hexadecane the microcosms were flushed with synthetic air containing oxygen and nitrogen but no carbon dioxide. An overview of the experimental design in given in Table 1. Pre-incubation and incubation were conducted at 12 °C, which is the average temperature of the groundwater in the pilot scale constructed wetlands, by slightly shaking the samples (20 rpm). Gas samples for CO_2_ concentration and δ^13^C measurements were collected throughout the experiment. The microcosms were incubated for 42 days. Then the water was decanted and the remaining filter material was analyzed for its TPH content and PLFA pattern.

### Design of the decane hydrogen isotope fractionation experiment

In the second experiment, we added decane and monitored its hydrogen stable isotope composition. The molecule decane was chosen because it is small enough to pronounce a measurable isotope effect, even if only 15–40% of added decane are biodegraded according to the fractionation factors given by Iannone et al. (2004) and Pond et al. (2002) and because decane represents the short chain and volatile compounds of diesel. Filter material samples for the microcosms experiments were collected from the CWs on the 18th of September 2014, after 728 days of operation. Between the first sampling in January for the hexadecane microcosms and the sampling in September 2014 sand + CW has received much less TPH and contained much less TPH than all the other CWs. Hence we decided to compare the three filter materials which had similar TPH loading, namely sand, expanded clay, and sand and biochar. The CWs had received around 256,000 mm of groundwater and 310 g of TPH (Supplementary material, Table 6). Sampling, transport and incubation temperature were identical to the hexadecane carbon isotope labelling experiment. The samples were analyzed for their TPH content. Five microcosms were set up per treatment, except for the sand & biochar treatment, where only 4 microcosms of two duplicated subsamples were incubated. Microcosms were pre-incubated for a minimum of 1 week and occasionally fed with decane. The water content of the filter materials was 9.2, 32.6 and 11.35 g per 100 g dry filter material for sand, expanded clay and sand and biochar, respectively. During pre-incubation and incubation oxygen concentration was monitored, and oxygen and macronutrients were replenished if necessary. For the incubation experiment, 15–40 µL of n-decane was injected to the microcosms and the decane concentration in the headspace and its δ^2^H value were analyzed with GC-IRMS. The injection volume was estimated from the diesel removal capacity of the pilot scale constructed wetland taking into account the date of sampling and that decane is a minor component in diesel in comparison to hexadecane. In the sand & biochar and expanded clay filters, decane was consumed quickly and replenished up to 6 times.

### Carbon stable isotope phospholipid fatty acid analysis

PLFA extraction was done according to the protocol of Watzinger et al. (2014) with a few adaptions. Firstly, 13:0 and 19:0 PLFAs standards were added with the methanol in the Bligh and Dyer extraction step. Secondly, to account for the specific conditions of the wetland filter material i.e. well buffered neutral to slightly base pH values and low amounts of PLFAs had to be implemented: the carbonate in the filter material was removed by adding 100 µL of 1.5 M HCl solution and 70 µL of 0.85 M HCl solution to 2 g of sandy material and expanded clay, respectively. After this treatment the phosphate buffer of the Bligh and Dyer solution was able to reach a constant pH value of 4.1 during PLFA extraction. To recover sufficient PLFAs for concentration and isotope ratio measurements 6 g of samples (three times 2 g) were extracted and combined to one sample after phase separation of the Bligh and Dyer solution. Measurements using a Gas chromatograph equipped with a flame ionization detector (GC-FID) and a GC coupled to an isotope ratio mass spectrometer via a combustion interface (GC-c-IRMS) were done according to Watzinger et al. (2014), except that the constant pressure of the Helium carrier gas in the GC-c-IRMS was kept at 195 kPa instead of 170 kPa. Peak attribution was done according to Watzinger and Hood-Nowotny (2019). The uptake of ^13^C hexadecane into total microbial PLFAs was calculated by using mass balance calculation as described by Watzinger (2015).

### Carbon stable isotope CO_2_ analysis

Measurement of the concentration and δ^13^C value of CO_2_ was done on a GC-c-IRMS according the method presented in Watzinger et al. (2014). Between 2.5 and 0.12 mL gas samples were injected twice and analysis was repeated if δ^13^C between replicate injections were > 0.5‰. CO_2_ standards ranging from 0.5 to 20 Vol‰ CO_2_ in He were used for calibration. The δ^13^C of the CO_2_ standard gas was determined on a EA-IRMS and referenced against certified international stable isotope standards. The reproducibility of the δ^13^C of the gas standard throughout the experiment was below 0.3‰ (single standard deviation). The amount of CO_2_ derived from the labelled hexadecane in percent was calculated by a simplified mass balance calculation (Supplementary material, Eq. 1).

### Total petroleum hydrocarbon analysis

Total petroleum hydrocarbon content was determined following the ISO procedure ISO16703:2004(E) (ISO 2004). Shortly, 10 mL of acetone were added to 10 g of filter samples and swung by hand. Afterwards 5 mL heptane containing 30 mg L^−1^ tetracontane and 30 µl L^−1^ decane was added and shaken for 1 h. The extraction suspension was washed twice with 60 mL of water. After centrifugation the upper organic phase was transferred into another tube and traces of water were removed by adding 1 g of Na_2_SO_4_. Florisil (MgO_3_Si) was added until the extract was clear. Water samples were extracted according to DIN EN ISO 9377-2:2001-07 (DIN 2001). Shortly, 50 mL heptane containing 30 mg L^−1^ tetracontane and 30 µL L^−1^ decane were added to 1 L of water and shaked for 30 min. Afterwards the organic and water phase was left to separate for 30 min. The organic phase was transferred into glass tubes containing 1 g of Na_2_SO_4_. Ten mL of extractant were concentrated in a rotavapor at − 120 mbar and 40–42 °C temperature to 1 mL. Measurement of both extracts was done by a gas chromatograph equipped with a flame ionization detector HP5890 (Hewlett Packard, Wilmington, USA). One µL sample was injected splitless and the split was opened between 1.5 and 20 min after injection. Separation was accomplished by a high temperature capillary GC column containing (5%-phenyl)-methylpolysiloxane (DB-5HT, 30 m × 0.25 mm, 0.1 µm; Agilent, USA). The carrier gas was He and the pressure was kept constant at 134 kPa. The injection temperature was 300 °C. The oven temperature was kept for 1 min at 60 °C and then raised at 20 °C min^−1^ to 340 °C, which was held for 10 min. The area between decane and tetracontane was integrated and reported as total petroleum hydrocarbons (TPH). Calibration between 10 and 4000 mg L^−1^ was done using a mineral oil standard mixture (Fluka, USA). Linearity was confirmed between 10–4000 mg TPH kg^−1^, recovery depended on TPH concentration and was 95%, 86% and 81% at 500, 1000 and 1660 mg TPH kg^−1^ respectively, the reproducibility was 1.2% relative standard deviation.

### Decane and hydrogen stable isotope analysis and calculations

Measurement of decane concentrations and hydrogen stable isotope values was conducted by a purge and trap unit (VSP4000, IMT GmbH, Vohenstrauß, Germany) coupled to a GC-IRMS. The gas chromatograph was a Trace GC and was coupled via a pyrolysis reactor and a Conflow IV to an isotope ratio mass spectrometer (Delta V advantage) (Thermo Fisher Scientific, Bremen, Germany). Decane in the headspace of the microcosms was transferred to a 20 mL headspace vial, which was purged with 20 mL min^−1^ He at 40 °C, for 20 min. After passing a Peltier water trap, decane was collected at a liquid nitrogen trap filled with a Tenax ® adsorbent at − 50 °C. It was desorbed and transferred for 50 s at 200 °C to the GC. The separation was done using a DB 624 capillary column (60 m × 0.32 mm × 1.8 µm). He flow rate was 1 mL min^−1^. The oven temperature was held for 4 min at 70 °C and consequently ramped to 190 °C at 20 °C min^−1^ and hold for 12.5 min. Conversion of decane into H_2_ was done using a high temperature conversion reactor (HTC oven) at 1420 °C. Referencing was done by measuring the decane (used as GC-IRMS standard and for the experiments) by a temperature conversion elemental analyzer coupled to an isotope ratio mass spectrometer (TC/EA-IRMS) against international certified stable isotope standards and lab standards as liquid injection and defined as δ^2^H_VSMOW_ = 81 ± 3‰. The uncertainty of the hydrogen stable isotope measurements was ± 5‰ for decane in groundwater samples and headspace samples. For the headspace standards and samples of this experiment precision and accuracy was excellent (δ^2^H = 81 ± 2‰). The lower method detection limit was 150 ng decane on column, reaching a 1 V peak amplitude. Consequently, aliquots of 30 to 2000 µL headspace samples from the microcosms were transferred into 20 mL vials samples to achieve 1—9 V peak amplitudes. The decane standard concentration was prepared in a mininert vial and different volumes were transferred into the headspace vial before measurement following the identical treatment principal (Werner and Brand 2001). Following the simplified Rayleigh equation, the bulk kinetic isotope effect expressed as enrichment factor ε_bulk_ was calculated as the slope of a linear regression line ln δ^2^H_bulk_ versus ln(f) (Supplementary material, Eqs. 2 and 3) (Scott et al. 2004). The enrichment factor on the reacting position ε_rp_ was calculated from the slope of a linear regression line ln δ^2^H_rp_ versus ln(f) and converted to AKIE (Supplementary material, Eqs. 4, 6 and 7) (Elsner et al. 2005).

### Statistical analysis

All results concentrations are reported as µg per gram dry filter material. Arithmetic means and standard deviations were calculated. Normality of distribution and homogeneity of variance were checked using Shapiro–Wilk-test and Kruskal–Wallis-test, respectively. For TPH and PLFA a one-way analysis of variances (ANOVA) (replaced by the Welch test, if homogeneity of variance was not achieved) followed by a post hoc test (Tukey test) was carried out to test the influence of the filter material using IBM SPSS 24. For the CO_2_ data, the factors time and filter material as well as their interaction were tested. Significance of difference was accepted for p ≤ 0.05. Additionally, principal component analysis was used to reduce the number of variables (single PLFA) for microbial community analysis. Significant differences between filter materials of the first two extracted principal components were tested by ANOVA. Correlation between TPH and total PLFAs was determined using the Pearson correlation coefficient (ρ). A linear regression for the different filter materials using CO_2_ production derived from hexadecane and decane concentration was calculated by SigmaPlot 14.0. The slope of the regression was reported as mineralization (time versus µg CO_2_ g^−1^ day^−1^) and the biodegradation rate of hexadecane and decane (day^−1^) was calculated with initial concentration set as 1.

## Results

### Total petroleum hydrocarbon and total microbial biomass

**Fig. 1:**
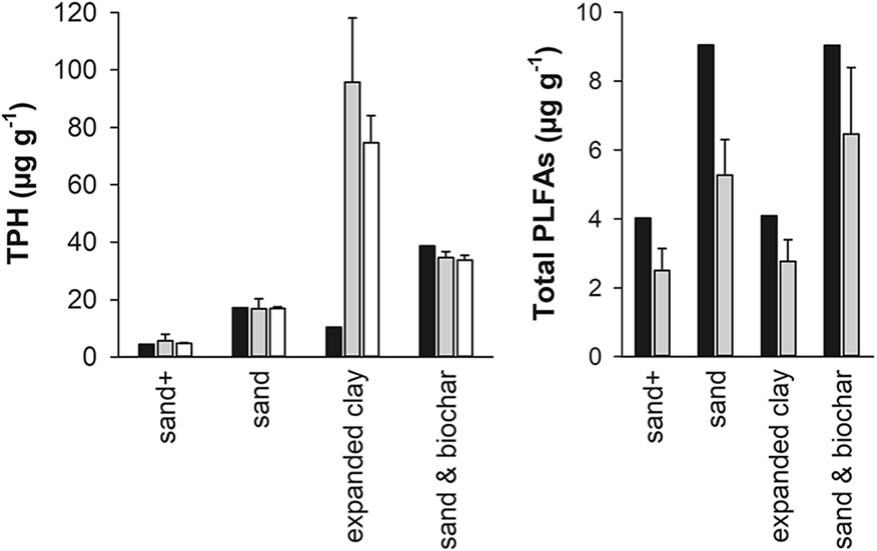
Total petroleum hydrocarbon (TPH) concentration (without hexadecane) in the wetland filter material before (black columns) (n = 1) and after incubation with hexadecane in active microcosms (gray columns) (n = 4) and in sterile controls (white columns) (n = 2). Total amount of microbial phospholipid fatty acids in the wetland filter material before (black columns) (n = 1) and after incubation in active microcosms (gray columns) (n = 4). The error bars represent single standard deviations

Individual samples of the CW had TPH concentrations ranging from 21 to 125 mg kg^−1^ TPH. A significant correlation between TPH and total microbial PLFAs concentrations (ρ = 0.89, p < 0.0001) was observed (Supplementary material, Fig. 1). At the end of the hexadecane carbon isotope labelling microcosms experiment the TPH (without the remaining ^13^C hexadecane) and the PLFA concentration was similar to the starting TPH and PLFA concentration except for TPH in the treatment with expanded clay (Fig. 1). There the TPH had increased nine-fold in both, the sterile and the living microcosms. This TPH concentration would amount to 7% of the added hexadecane. However, the hydrocarbon peaks which contributed to the TPH increase did not possess an elevated δ^13^C value i.e. apparently were not derived from the added ^13^C hexadecane. Additionally, we tested if the addition of hexadecane increased the extractability of non-hexadecane TPH, by adding hexadecane a few hours before extraction was commenced. Results showed no impact of hexadecane on the TPH extractability in the short term. Also grinding the expanded clay to release the inner surface did not increase TPH extraction. It appeared that only the long term (42 days) addition of hexadecane onto expanded clay had increased the extractable TPH. This TPH increase in expanded clay was not mirrored in the total microbial PLFA concentrations (Fig. 1).

### Mineralization of hexadecane

**Fig. 2:**
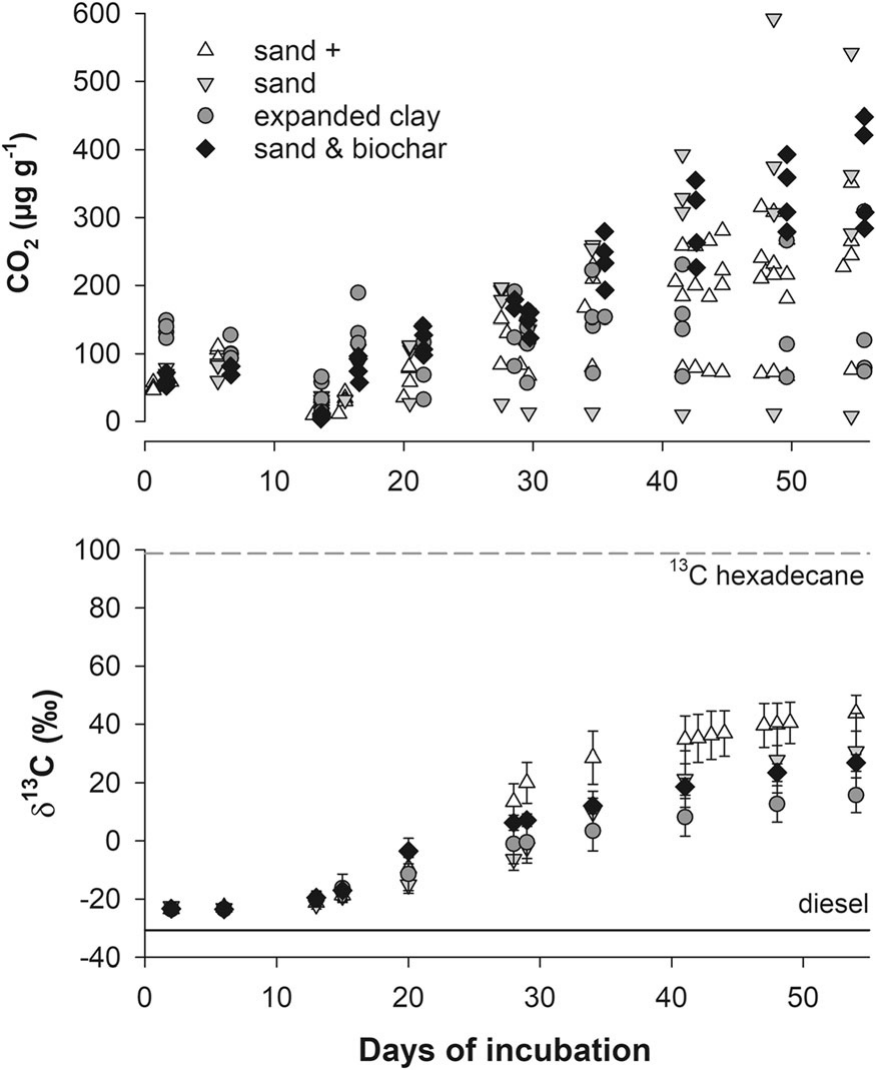
Concentration (single values) and δ^13^C values of the CO_2_ in the microcosms. The error bars represent single standard deviations, the dashed gray lines indicate the δ^13^C value of the labelled hexadecane added on day 12 and the full black line the δ^13^C value of the diesel previously applied to the constructed wetlands

Before the labelled hexadecane was added, the CO_2_ production was around 100 µg CO_2_ g^−1^ dry filter material and its δ^13^C value was − 23.2‰ (Fig. 2). In comparison, the diesel which had been added to the CWs for several months before filter material sampling had a δ^13^C value of − 30.7 ‰. After ^13^C hexadecane was added, the CO_2_ increased. Variation in CO_2_ production between replicate microcosms was remarkable. Still, CO_2_ production was higher in the sand and biochar compared to the expanded clay. Even though replicates had produced very different CO_2_ concentration, their δ^13^C CO_2_ values varied little and the influence of the filter material on the δ^13^C CO_2_ value was highly significant (Supplementary material, Table 7). At the end of incubation 55% (sand +), 44% (sand), 32% (expanded clay) and 41% (sand & biochar) of the released CO_2_ had their origins in the ^13^C hexadecane. The CO_2_ concentrations and δ^13^C CO_2_ value in the sterile controls remained constant (4 ± 2 µg CO_2_ g^−1^ dry filter, δ^13^C CO_2_ value = − 20 ± 3 ‰).

**Fig. 3:**
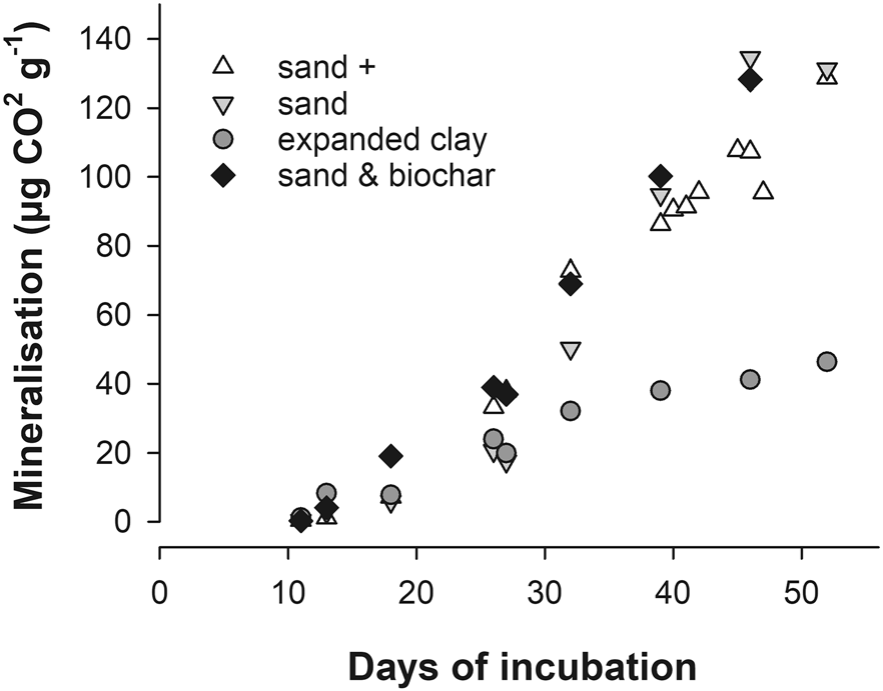
Mineralization of hexadecane

Using the amount of produced CO_2_ and the fraction derived from hexadecane biodegradation, we were able to calculate the hexadecane mineralization in µg CO_2_ g^−1^ (Fig. 3). The mineralization rate of hexadecane in expanded clay was three times lower than in the sandy filter materials (Table 2). The hexadecane mineralization was linear, i.e. it followed a zero order kinetic. The complete apparent biodegradation rate of hexadecane was calculated by relating mineralized hexadecane to its input concentration. We found biodegradation rates of hexadecane in sandy filters of − 0.0014 day^−1^, while the biodegradation rate of expanded clay was lower (− 0.0003 day^−1^) (Table 2).

**Table 2:** The mineralization and the degradation rate (k) of n-hexadecane ± standard deviation and the coefficient of determination (R^2^) for combined data (n = 4) are reported (T = 12 °C, 1 µL hexadecane/g substrate, water-saturated). The flow of hexadecane into the pools CO_2_ (i.e. mineralization), microorganisms and total petroleum hydrocarbon other than hexadecane after 42 days of incubation was calculated. The amount of remaining carbon (as hexadecane or other metabolites) is reported

Filter material	Sand +	Sand	Expanded clay	Sand & biochar
Mineralisation^a^ (µg CO_2_ g^−1^ day^−1^)	3.2 ± 0.4	4.0 ± 0.7	1.0 ± 0.2	3.7 ± 0.2
Degradation rate^a^ k (day^−1^)	− 0.0012 ± 0.0001	− 0.0016 ± 0.0003	− 0.0003 ± 0.00005	− 0.0014 ± 0.00007
R^2^	0.58	0.50	0.67	0.91
Distribution (%)				
CO_2_—C	4.9	5.4	1.2	5.5
Microbial—C	1.8	2.3	2.8	3.8
TPH—C^b^	0	0	0	0
Remaining—C	93	92	96	91

^a^Zero order kinetic

^b^Total petroleum hydrocarbons without hexadecane

### Microbial community characterization and hexadecane anabolisation

**Fig. 4:**
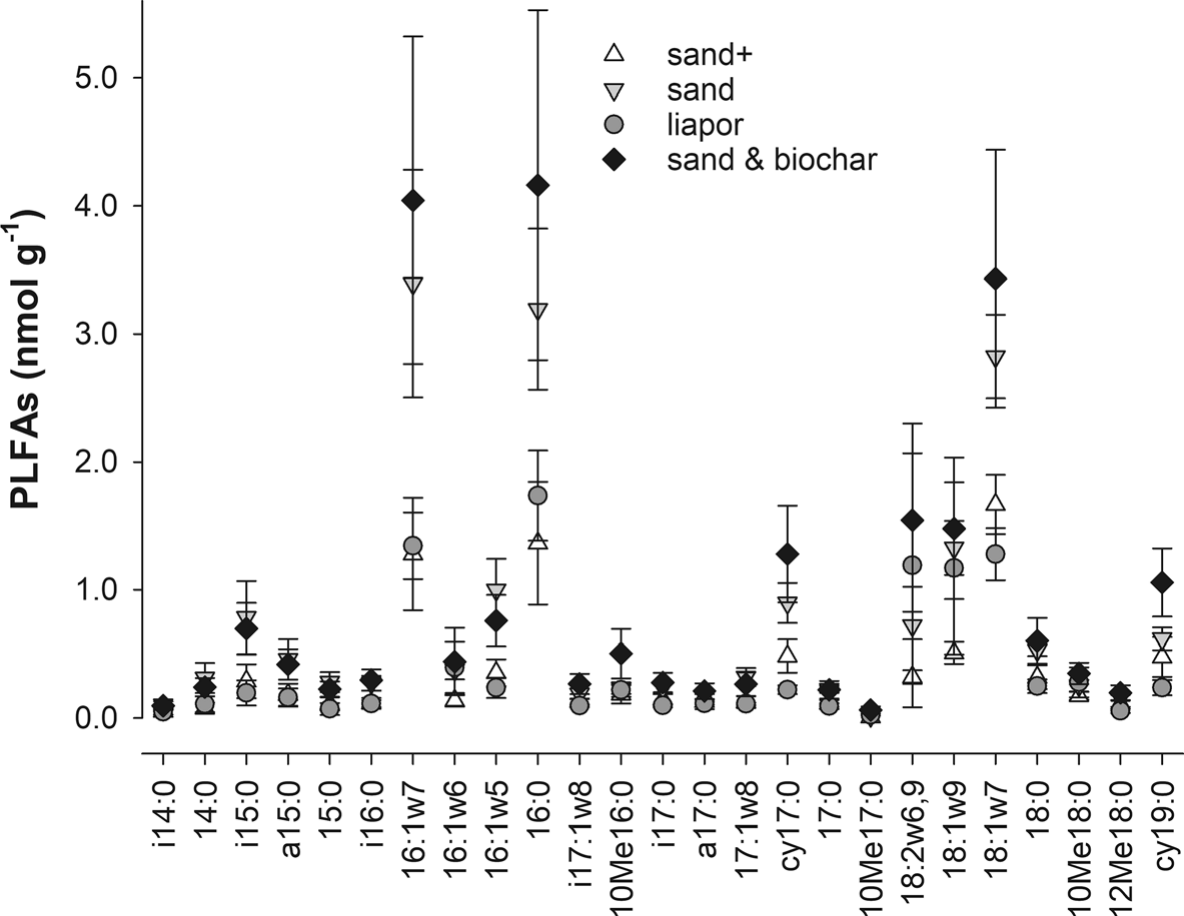
Concentration of the single microbial phospholipid fatty acids (PLFAs) in the living microcosms (n = 4). The error bars represent single standard deviations

The PLFAs were extracted before and after 42 days’ incubation period. PLFA biomarkers of all microbial groups (Gram + bacteria, Gram- bacteria, actinomycetes and fungi and were present in the constructed wetland samples, but the microbial PLFA pattern was dominated by 16:1ω7c (23%), 16:0 (16%), 18:2ω6,9c (7%), 18:1ω9c (8%) and 18:1ω7c (10%) (Supplementary material, Figs. 2 and 3). During incubation this general PLFA pattern did not change (Fig. 4). The microbial community was significantly influenced by the filter material used for remediation. Single PLFAs and the first factor after principal component analysis (PCA) were significantly impacted (Supplementary material, Fig. 4). The impact of filter material was already visible in the PLFA pattern analyzed straight after collection from the CW and even more pronounced after material homogenization and incubation (Supplementary material, Fig. 4).

**Fig. 5:**
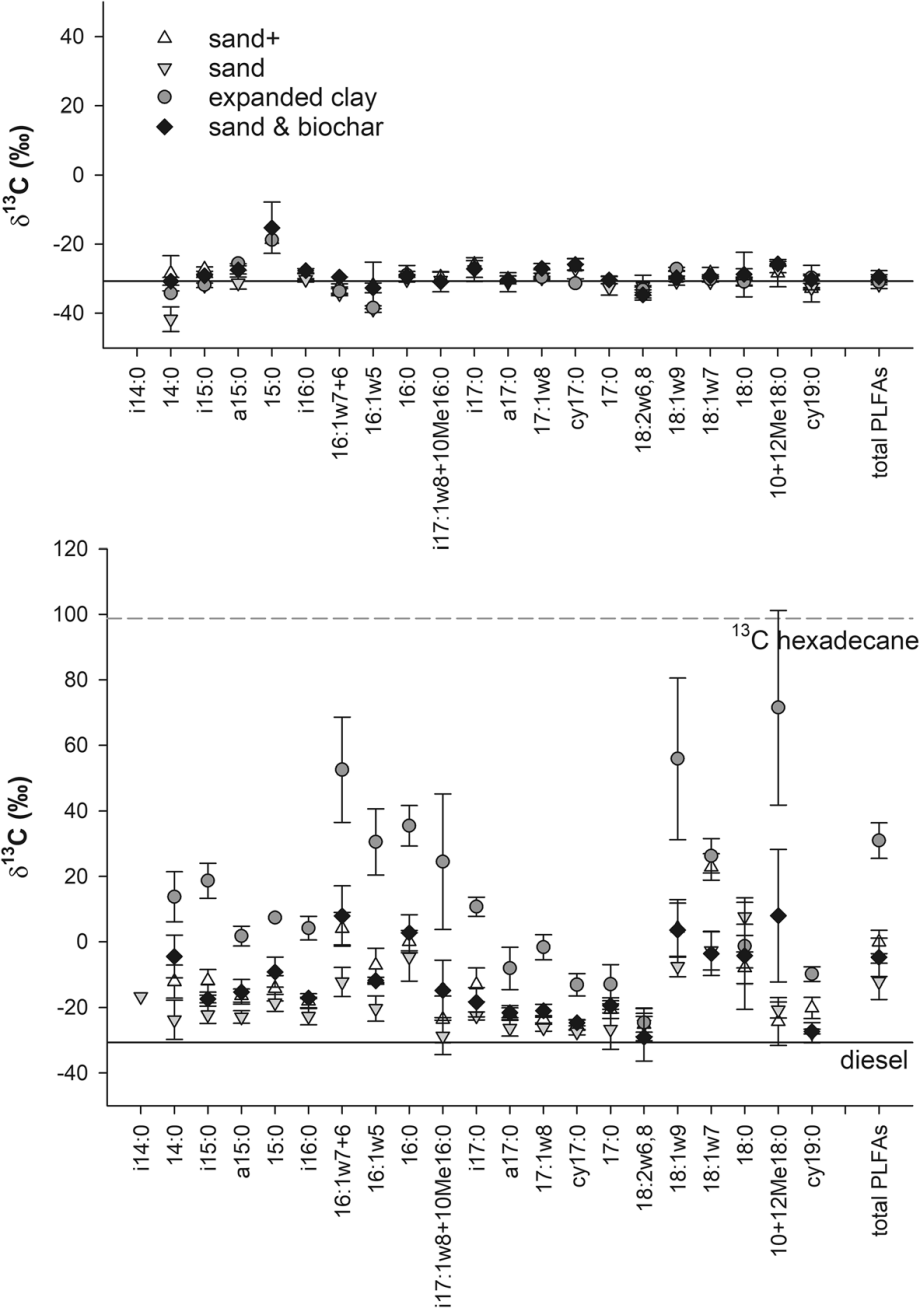
The δ^13^C values of the single and total fatty acid methyl esters of microbial phospholipid fatty acids in the sterile wetland filter materials (n = 2) (upper graph) and the living microcosms (lower graph) (n = 4). The error bars represent single standard deviation, the dashed gray lines indicate the δ^13^C value of the labelled hexadecane and the full black line the δ^13^C value of the diesel previously applied to the constructed wetlands

Some microbial PLFAs were extracted from the sterile controls but at low amounts. The ^13^C PLFA signature of the sterile controls was close to the δ^13^C of the diesel (− 30‰) which had been applied to the constructed wetlands several months before sampling. Only the δ^13^C of the PLFA 15:0 was elevated by 12‰ (Fig. 5), possibly due to small amounts of ^13^C hexadecane co-extracted with PLFAs. All individual PLFAs in the living microcosms had significantly elevated δ^13^C. The most prominent difference was the higher ^13^C label in expanded clay in comparison to the sandy filter materials. The distribution of the label was rather uniform in contrast to the microbial community structure. The PLFAs with the highest label fraction were 16:1ω6c&7c, 18:1ω9c, 18:1ω7c, 16:0 and 10&12Me18:0. In contrast, the fungal biomarker 18:2ω6,9 were least labelled.

The carbon isotope discrimination between filter material and PLFA was determined as − 0.3‰ on CW samples fed with diesel and before the labelled experiment commenced. The discrimination was considered when calculating the uptake of hexadecane into PLFAs. The percentages of PLFAs derived from hexadecane were 24%, 15%, 48% and 20% for the sand + , sand, expanded clay and sand & biochar filter materials, respectively.

### Pool size distribution of hexadecane

The flow of hexadecane after 42 days into the various pools, CO_2_, microbial and TPH (without hexadecane) was calculated as percent carbon derived from hexadecane (Table 2). For converting the amount of PLFA (nmol g^−1^) into microbial carbon (µg g^−1^), the conversion factor of 5.8 was used (Joergensen and Emmerling 2006). The percentage of remaining hexadecane was calculated by adding up to 100%. In sandy filter materials around 5% of the added hexadecane was mineralized. 1.8–3.8% carbon was stored in the microbial biomass. Less hexadecane was mineralized in the expanded clay (1%). Additionally, a release of 7% non-hexadecane hydrocarbon in both sterile and non-sterile expanded clay was observed after 42 days.

### Decane degradation and hydrogen isotope fractionation

**Fig. 6:**
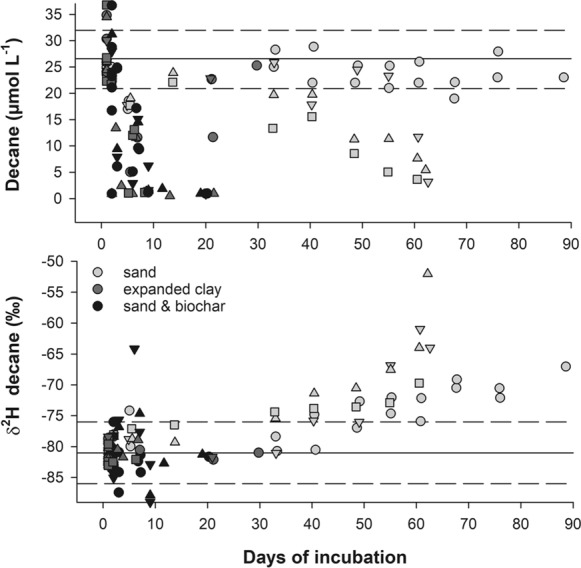
Concentration and δ^2^H value of decane in the headspace of the microcosms. Decane was added to the CW filters in surplus (15–40 µL) and concentration in the headspace only decreased after liquid decane was exhausted. Solid lines indicate the decane starting concentration (in equilibrium with the liquid phase) and its δ^2^H value, dashed lines indicate measurement uncertainty. Different colors and symbols illustrate the different treatments and replicates, respectively

Liquid decane was added in surplus and we expected that stable isotope fractionation in the headspace was only visible at a later stage of biodegradation when liquid decane became exhausted. The bottles with gaseous decane standard as well as the sterile controls contained a decane concentration of 26.6 ± 5.6 µmol L^−1^ decane at 12 °C throughout the incubation time. Applied decane was used up faster in expanded clay and sand & biochar microcosms than in the pure sand filters. In expanded clay and sand & biochar filters, the greatest challenge was to sample during the short time interval when decane concentration in the headspace was decreasing. This small sampling window was only open for a few hours. Most of these time points revealed that the residual fraction of decane was not enriched in deuterium in the expanded clay and four slightly enriched samples (δ^2^H value was outside the measurement uncertainty) were found in sand & biochar filters (Fig. 6).

In the sand filters, decane concentrations of two replicates did not decrease in the headspace during the incubation period of 90 days, but decane in the headspace got clearly enriched, indicating bond cleavage (Fig. 6). The biodegraded decane is mixed with the non-biodegraded decane pool and the remaining fraction f cannot be determined. Fortunately, in three sand filter replicates decane concentrations decreased in the headspace. The Rayleigh equation can be applied (Khan et al. 2018) and data from the point on when decane in the headspace started to decrease were used for calculation of the apparent hydrogen kinetic isotope effect (Table 3 and Supplementary material, Table 7). Zero order kinetic gave the best fit for the biodegradation rates and the arithmetic mean and standard deviation was -0.036 ± 0.027 day^−1^ (Table 3). The rate and variability was likely underestimated because the two low degrading sand replicates were not included.

**Table 3:** AKIE and apparent biodegradation rates (k) for three sand microcosm replicates (T = 12 °C, 1 µL decane/g filter material, water-unsaturated)

	Sand 3	Sand 4	Sand 5
AKIE_H_	1.129	1.500	1.072
k (day^−1^)	− 0.067	− 0.025	− 0.017

## Discussion

We investigated the petroleum hydrocarbon biodegradation in three different constructed wetland filter materials using two model substances, decane and hexadecane, and two different isotope techniques. Both compounds were applied to the filter materials as a pure non-dissolved phase. This was appropriate, because we also expected, that in highly contaminated groundwater due to the low solubility of hydrocarbons in water (10^–5^ mg L^−1^ for hexadecane, 0.02 mg L^−1^ for decane at 25 °C (Mackay et al. 2006)), compounds would be present as non-aqueous phase solutions. Starting with the non-dissolved phase we expected partitioning in the different compartments (air, water, filter material), which would strongly influence the bioavailability and hence our biodegradation efficiency.

### Mass transfer limiting decane biodegradation

Biodegradation was confirmed for decane and hexadecane. Theoretical kinetic hydrogen isotope effects derived from the transition state theory Streitwieser limit (KIE_H_ = 3.2) (Yoshizawa 2002) and apparent kinetic isotope effects found experimentally (AKIE_H_ = 2.632; AKIE_H_ = 2.263 derived from n-C_16_; AKIE_H_ = 1.306 derived from n-C_15_) (Pond et al. 2002; Iannone et al. 2004) were calculated after eliminating the dilution and intramolecular competition effects for the activation mechanism of terminal methyl group oxidation during biodegradation of decane (Elsner et al. 2005). The apparent kinetic isotope effects (AKIE_H_) seen for decane in our experiment was below the values expected for the intrinsic KIE, however within the range observed by Pond et al. (2002). If the observed (apparent) stable isotope fractionation factor is smaller than the one expected from bond cleavage, then other processes might limit biotransformation, such as dissolution or mass transfer (Kampara et al. 2008; Elsner 2010; Thullner et al. 2013). Decane volatilization after addition is instant, vapor phase transport is also expected to be fast, as demonstrated by Pasteris et al. (2002). We propose, that solubilisation and transfer in water (mainly driven by diffusion as the bottles were only swung before headspace sampling) limited bioavailability. Hence mass transfer of decane to the reactive site likely masked isotope fractionation and limited biodegradation. Additionally, back reporting of isotopically enriched decane from the vicinity of the enzymatic breakdown to the gas phase was largely absent in expanded clay and sand & biochar filter materials. This indicates that decane concentrations at the enzyme was either low, or that back diffusing decane was held back by sorption onto filter particles. Both processes might have occurred. Sand & biochar and expanded clay likely have a higher sorption capacity for organic contaminants than pure sand (Dordio et al. 2007; Bushnaf et al. 2011; Rossi et al. 2013). In our batch experiment, expanded clay and sand & biochar biodegraded decane faster than sand and we were also able to show, that microbial biomass was positively correlated to extractable TPH concentrations in the filters. This implies, that sorption was not the major factor retarding decane biodegradation, possibly because we had a very well adapted microbial community (728 days of CW operation), which was apparently able to compete successfully for decane. Reported negative impacts on biodegradation due to sorption was related to “sorption unadapted” microbial communities (Bushnaf et al. 2017). Fast biodegradation will further increase the relevance of mass transfer thus masking isotope fractionation.

In comparison to decane biodegradation rate from soil lysimeter studies (− 5.0 day^−1^) (Pasteris et al. 2002), column studies (− 5.8 day^−1^, − 13.5 day^−1^,) (Höhener et al. 2003, 2006) and batch studies (− 1.4 day^−1^, − 1.2 day^−1^) (Höhener et al. 2006; Birch et al. 2017) our rates of the pure sand (− 0.036 day^−1^) calculated as zero order kinetic from the headspace were two magnitudes lower, additionally supporting the assumption that biodegradation was mass transfer limited, and we therefore rather suggest using the wording apparent biodegradation rate. The variability of the biodegradation rate between replicates was high which reflects the inhomogeneity of the filter material. The observed small biodegradation rate of decane in combination with high vapor phase transport in sand as observed by Pasteris et al. (2002) might lead to a substantial loss via volatilization in constructed wetlands. However, we expect that in the constructed wetland especially under intermittent loading operation, periodical flooding, drainage and intrusion of air will likely foster biodegradation due to mixing, while our microcosms decane degraders had to rely on diffusive contaminant transport only.

### Hexadecane biodegradation and the impact of sorption in expanded clay filters

Hexadecane biodegradation was confirmed by production of labelled CO_2_, i.e. microbial mineralisation and labelling of microbial biomembranes. The mineralisation rate was lower than those of various soils (Freijer et al. 1996). Accordingly, our apparent biodegradation rate in sandy filter material (− 0.0014 day^−1^) and expanded clay (− 0.0003 day^−1^) was also lower than cited biodegradation rates (− 0.0281 day^−1^ in activated sewage sludge (Pond et al. 2002)). Removal rates of constructed wetlands were up to four magnitudes higher (-0.1 to − 2.5 day^−1^ for oil and grease (Horner et al. 2012; Alley et al. 2013), − 6 to − 30 day^−1^ for n-alkanes (Salmon et al. 1998), − 0.008 to − 0.0236 day^−1^ for diesel calculated as first order reaction from the removal data presented by Al-Baldawi et al. (2013)) than observed in our microcosms study. Due to its length, hexadecane is less soluble in water than decane, which is reflected when comparing biodegradation rates. It needs to be stated though, that comparison of biodegradation rates might be misleading because we determined hexadecane biodegradation as total mineralization to CO_2_ while for decane and in many other studies it is defined as loss of the mother compound. Hence we might have underestimated the hexadecane biodegradation rate. Assuming that the first oxidation on the terminal or subterminal carbon (one carbon out of 16) would already lead to vanishing of the mother compound and that around 60% of the biodegraded carbon was released as CO_2_ in 42 days while the remaining was stored in the microbial biomass (Table 2), we expected biodegradation rate of to − 0.008 to − 0.037 day^−1^, which were closer to the values reported in literature, especially when considering that our study was conducted at a temperature of 12 °C. At 12 °C, the added hexadecane was visible as a viscous droplet on the surface of the filter material for several days. Dissolution of the hexadecane into the water, transport through the water, and sorption on the filter material surface all will follow zero order kinetics, which we observed for mineralisation. Microbial biomass did not increase during incubation, even though higher mineralisation rates during preincubation revealed a higher biodegradation potential of the microbial community. Nutrients and oxygen composition were monitored and limitation was avoided by addition of oxygen and nutrients, thus we assumed sufficient supply. We can conclude that low biodegradation rates, the lack of bacterial growth and zero order biodegradation kinetics indicated that the bioavailability of hexadecane controlled biodegradation. Also in bioreactor systems (with agitation resulting in smaller hexadecane droplet sizes) hexadecane biodegradation was transfer limited (Quijano et al. 2010; Lizardi-Jimnez et al. 2011).

Interestingly, mineralisation of hexadecane was lower in the expanded clay in comparison to sandy material. This can only partly be attributed to lower microbial biomass and activity. The δ^13^C value of the CO_2_ indicated that hexadecane was competing with other carbon sources, mainly TPH sorbed onto the filter during former CW operation. Competition was lower in sand with low TPH concentrations (55% of CO_2_ derived from hexadecane) than in expanded clay with only 32% CO_2_ deriving from hexadecane. In the expanded clay we also extracted high amounts of hydrocarbon formerly applied to the constructed wetland. Apparently expanded clay has the ability to adsorb hydrocarbons, which then are slowly released after hexadecane addition. In accordance, Machado et al. (2017) observed sorption kinetics in expanded clay lasting over several days, but with high final removal rates. Sorption by expanded clay appeared less relevant when decane was added, possibly because decane is more soluble and less hydrophobic. It was found recently, that adsorption to expanded clay increases with the hydrophobicity of crude oil (Pouramini et al. 2019).

### Established microbial community and involvement in hexadecane biodegradation

In accordance with current knowledge (Rojo 2009; Abbasian et al. 2015a), hexadecane was consumed by most microbial groups as the hexadecane label was distributed throughout bacterial PLFAs. We are aware that secondary turnover might also lead to some labelling of microbial PLFAs which are not directly involved in the biodegradation of the respective compound (Mellendorf et al. 2010; Wawra et al. 2018). If only secondary turnover is responsible this usually leads to low label uptake in non-degrading microorganisms, which was only observed for the fungal biomarker (18:2ω6,9) in this study. In contrast, many fungi are known to biodegrade hexadecane and some use hydrocarbons as a sole carbon and energy source (Prenafeta-Boldú et al. 2019). Some polycyclic aromatic hydrocarbons (Johnsen et al. 2002; Mellendorf et al. 2010) and few hexadecane (Adetutu et al. 2012) biodegradation studies however have shown that fungi can be involved in the first reactions possibly using nonspecific extracellular enzymes without further mineralizing and incorporating the compound. We have likely observed a similar behavior.

In contrast to uptake, the microbial community in the wetland was dominated by a few PLFAs, belonging to Gram- bacteria (monounsaturated FA: 16:1ω7c, 18:1ω7c) and fungi (18:2ω6,9 and 18:1ω9c). 16:0 is ubiquitous and present in high amounts of Gram- bacteria. The bacteria (16:1ω7c, cy17:0, 18:1ω7c, cy19:0, i14:0, i15:0, a15:0, i16:0, i17:0, a17:0) to fungi (18:2ω6,9) ratio was around 14 ± 2 in the pure sandy filters and 7 ± 3 in the expanded clay and sand & biochar filters. Both expanded clay and biochar seemed to present a better habitat for fungi, possibly due to their lower bulk density / higher pore volume. We even observed mycelia in the expanded clay filters. In comparison to soil habitats the PLFA “diversity” is poor, but it compares to former studies of constructed wetland, where domination of Gram- bacteria and fungi and low abundance of actinomycetes and Gram + bacteria was seen (Jin and Kelley 2007; Tietz et al. 2008). The microbial communities had adapted to the filter material. We suggest that the microbial community is selected by the environmental conditions in the constructed wetland rather than their ability to biodegrade hydrocarbons; an ability which confirmed widespread among the bacteria and actinomycetes. The microorganisms also feed on non labelled TPH. Interestingly in expanded clay, microorganisms were incorporating more hexadecane in respect to other carbon sources after 42 days. At the moment there is no firm explanation for this behavior. Possibly initial sorption processes reduced bioavailability but after 42 days hexadecane became bioavailable, which has also been observed for pharmaceuticals and polycyclic aromatic hydrocarbons by Dordio et al. (2010), Machado et al. (2017) and Nkansah et al. (2012).

## Conclusion

Observed microbial biodegradation of decane and hexadecane was lower in comparison to published lab and pilot scale experiments, and mass transfer likely limited biodegradation. This implies, that in a full scale constructed wetland bioremediation plant, measures to improve the contact between TPH and the filter material such as intermittent loading and subsurface irrigation to reduce volatilization of short chain hydrocarbons might be beneficial and are strongly recommended. With respect to the choice of the filter material, expanded clay might retain petroleum hydrocarbons, however, slow release might prolongate biodegradation. Sand amended with biochar successfully supported the biodegradation of short (decane) and middle chain hydrocarbons (hexadecane) and can be recommended as filter material in CWs.

## Supplementary Information

Below is the link to the electronic supplementary material.

Supplementary file1 (PDF 633 kb)

## Data Availability

The datasets generated during the current study are available from the corresponding author on reasonable request.
