# Supplementary material for: Unravelling the process of petroleum hydrocarbon biodegradation in different filter materials of constructed wetlands by stable isotope fractionation and labelling studies
Source: Biodegradation. 2021 Apr 16;32(3):343–59. doi: 10.1007/s10532-021-09942-1 (PMC8134294; doi:10.1007/s10532-021-09942-1)
Supplement: Supplementary file 1 — Supplementary file1 (PDF 633 kb) [file 10532_2021_9942_MOESM1_ESM.pdf]

Electronic Supplementary Material for Biodegradation

Unravelling the process of petroleum hydrocarbon biodegradation in different filter materials of constructed wetlands by stable isotope fractionation and labelling studies

Andrea Watzinger[a,b] *, Melanie Hager[a,b], Thomas Reichenauer[b,c], Gerhard Soja[b,d], Paul Kinner[b]

[a] *Institute of Soil Research, Department of Forest- and Soil Sciences, University of Natural Resources and Life Sciences, Konrad Lorenz-Strasse 24, 3430 Tulln, Austria,*
[b] *Environmental Resources & Technologies, Energy Department, AIT – Austrian Institute of Technology GmbH, Konrad Lorenz-Strasse 24, 3430 Tulln, Austria*
[c] *Bioresources, Center of Health & Bioresources, AIT – Austrian Institute of Technology GmbH, Konrad Lorenz-Strasse 24, 3430 Tulln, Austria*
[d] *Institute for Chemical and Energy Engineering, Department of Material Sciences and Process Engineering, University of Natural Resources and Life Sciences, Muthgasse 107, 1190 Vienna, Austria*

** Corresponding author: andrea.watzinger@boku.ac.at*

Table S1: Denomination, filter material and hydraulic characteristics of the constructed wetlands

Parameter	sand+	sand	expanded clay	sand & biochar
Filter material	quartz sand (washed)	quartz sand (washed)	expanded clay (Liapor HD, closed)	quartz sand (washed) + 3 w/w % biochar
Particle size	0-4 mm	0-4 mm	1-4 mm	0-4 mm
Minimum hydraulic loading rate (mm day^{-1})	67	70	56	60
Maximum hydraulic loading rate (mm day^{-1})	749	750	852	751
Average hydraulic loading rate (mm day^{-1})	441	352	404	387
Average hydraulic retention time ($days^{-1}$) [1]	5.5	7.3	6.3	6.4

[1] at the lowest 35 cm of the filter & drainage layer.

Table S2: Characteristics of the expanded clay: Liapor HD 1/4

Chemical components	Amount
SiO_2 (weight %)	60 ± 5
Al_2O_3 (weight %)	20 ± 5
Fe_2O_3 (weight %)	10 ± 5
CaO (weight %)	5 ± 5
Trace elements (weight %)	5 ± 5
Physical characterisation	
bulk density (kg m^{-3})	1 250 ± 50
apparent density (kg m^{-3})	750 ± 50
Water content (weight %) [1]	8 ± 4
Particles <1 mm (weight %)	< 1
Particles 1-2 mm (weight %)	10
Particles 2-3 mm (weight %)	50
Particles 3-4 mm (weight %)	40

[1] after 60 min

Table S3: Characteristics of the biochar "Sonnenerde"

Chemical components	Amount
pH (H_2O)	8.97
pH ($CaCl_2$)	8.78
TC (weight %)	63.2
TOC (weight %)	63.2
Total N content (g kg^{-1})	14.6
Total P content (g kg^{-1})	5.7
Ca (g kg^{-1})	69.78
Mg (g kg^{-1})	2.97
K (g kg^{-1})	14.51
Na (g kg^{-1})	0.46
PCB sum (7) (mg kg^{-1})	< 0.12
PCDD/F (WHO 2005) (ng kg^{-1})	0.905
PAH sum (16) (mg kg^{-1})	1.6
Physical characterisation	
WHC (weight %)	299
BET Oberfläche (ISO 9277) (m^2 g^{-1})	69.1

TC = Total carbon content

TOC = Total organic carbon

PCB = Polyclorinated biphenyl

PCDD/F = Polychlorinated dibenzodioxine and dibenzofurane

PAH = Polycyclic aromatic hydrocarbons

WHC = Water holding capacity

Table S4: TPH and PLFA concentrations (arithmetic means and standard deviations, n = 5) of the CW filter material and loads received on the day of sampling after 478 days operation (15th January 2014) and one month prior to sampling. The filters were used for the hexadecane carbon isotope labelling study.

Parameter	sand+	sand	expanded clay	sand & biochar
TPH (µg g^{-1})	39 ± 4	59 ± 12	86 ± 31	68 ± 31
Total PLFAs (µg g^{-1})	4 ± 2	10 ± 4	15 ± 9	4 ± 4
Total hydraulic loads (mm) after 478 days	138 446	95 066	146 592	113 439
Total TPH load (g m^{-2})	62.4	42.8	80.0	55.6
TOC load (g m^{-2})	2 381	1 635	2 521	1 951
Total inorganic N load (g m^{-2})	82.7	56.8	87.6	67.8
Total dissolved PO_4-P load (g m^{-2})	2.1	1.4	2.2	1.7
Total K load (g m^{-2})	1 391	955	1 473	1 139
Total TPH removal efficiency (%)	82.7	83.8	83.7	87.2
Monthly hydraulic loads (l m^{-2}) [1]	16 166	11 182	13 583	9 782
TPH (mg l^{-1}) [1]	0.79	0.79	0.79	0.79
Monthly TPH loads (g m^{-2}) [1]	12.8	8.8	10.8	7.6

[1] 30 days before sampling

TPH = Total petroleum hydrocarbon

TOC = Total organic carbon

Table S5: Hydrochemical characterisation of the groundwater used for the incubation experiment.

Parameter	Concentration [mg L^{-1}]
Ca	210
Fe	0.61
K	9.1
Mg	57

Mn	1.5
Na	57
NO_3	3.5
SO_4	177
PO_4	0.01
NH_4	0.25
NO_2	0.24
TOC	15

TOC = Total organic carbon

Table S6: TPH concentrations (arithmetic means and standard deviations, n = 5) of the CW filter materials and loads on the day of sampling after 728 days operation (28th of September 2014) and one month prior to sampling. The filters were used for the decane hydrogen isotope fractionation study.

Parameter	sand+	sand	expanded clay	sand & biochar
TPH (µg g^{-1})	35 ± 27	267 ± 151	113 ± 106	153 ± 63
Total hydraulic loads (mm) after 728 days	271 925	231 289	273 448	263 763
Total TPH load (g m^{-2})	128	311	279	342
TOC load (g m^{-2})	4 326	3 680	4 350	4 138
Total inorganic N load (g m^{-2})	287	244	288	278
Total dissolved PO_4-P load (g m^{-2})	4.4	3.7	4.4	4.2
Total K load (g m^{-2})	2 639	2 244	2 653	2 559
Total TPH removal efficiency (%)	77.2	91.9	89.9	93.7
Monthly hydraulic loads (l m^{-2}) [1]	20 349	17 543	15 034	17 456
TPH (mg l^{-1}) [1]	0.17	1.90	1.73	1.90
Monthly TPH loads (g m^{-2}) [1]	3.5	33	26	33

[1] 30 days before sampling

TPH = Total petroleum hydrocarbon

TOC = Total organic carbon

Calculating the percent CO_2 derived from hexadecane

$$C_{C16}(\%) = \frac{(\delta^{13}\mathrm{C\ CO_2}_{\mathrm{\ filter\ +C16}} - \delta^{13}\mathrm{C\ CO_2}_{\mathrm{\ filter}})}{(\delta^{13}\mathrm{C\ CO_2}_{\mathrm{\ C16}} - \delta^{13}\mathrm{C\ CO_2}_{\mathrm{\ filter}})} \times 100 \qquad \text{Eq. 1}$$

where $\mathrm{C_{C16}}(\%)$ is the fraction of carbon derived from hexadecane, $\delta^{13}\mathrm{C\ CO_2}_{\mathrm{\ filter\ +C16}}$ the $\delta^{13}C$ value of the CO_2 emitted from the hexadecane amended filters, $\delta^{13}\mathrm{C\ CO_2}_{\mathrm{\ filter}}$ the $\delta^{13}C$ value of the CO_2 released from the filter before labelling and $\delta^{13}\mathrm{C\ CO_2}_{\mathrm{\ C16}}$ the $\delta^{13}C$ value of the hexadecane.

Calculating the apparent kinetic isotope effect

The bulk kinetic isotope effect expressed as enrichment factor ε_{bulk} was calculated as the slope of a linear regression line ln δ^2H_{bulk} versus ln(f) (Eq 1, Eq 2).

$$\ln \delta^2\mathrm{H}_{bulk} = \ln\left(\frac{1000+\ \delta^2\mathrm{H}_t}{1000+\delta^2\mathrm{H}_0}\right) \qquad \text{Eq. 2}$$

$$\ln f = ln\frac{c_t}{c_0} \qquad \text{Eq. 3}$$

where c_0 and δ^2H_0 are the concentration and the δ^2H value in ‰ of the biodegraded substrate decane at the time point zero (start of degradation experiment) while c_t and δ^2H_t are the concentration and the δ^2H value of the degradation substrate decane at time points measured during the degradation experiment. f is the fraction remaining after degradation of the initial concentration c_0.

The enrichment factor on the reacting position ε_{rp} was calculated from the slope of a linear regression line ln δ^2H_{rp} versus ln(f). To gain the δ^2H on the reactive position δ^2H_{rp} the increase of the δ^2H value denoted as Δ^2H was corrected for the number of hydrogen in the substrate (decane; n = 22) divided by the number of hydrogen located at the reactive site (decane; x = 6) (Eq 3 and 4). ε_{rp} was converted into AKIE by accounting for the intramolecular isotopic competition (Eq 5) (decane; z = 6) (Elsner et al. 2005).

$$\Delta^2\mathrm{H}_t = \delta^2\mathrm{H}_t - \delta^2\mathrm{H}_0 \qquad \text{Eq. 4}$$

$$\ln \delta^2\mathrm{H}_{rp} = ln\left[\frac{1000+(\delta^2\mathrm{H}_0 + {}^{n}/_{x} + \Delta^2\mathrm{H}_t)}{1000+\delta^2\mathrm{H}_0}\right] \qquad \text{Eq. 5}$$

$$AKIE = \frac{1}{(1+\varepsilon_{rp}\cdot z)} \qquad \text{Eq. 6}$$

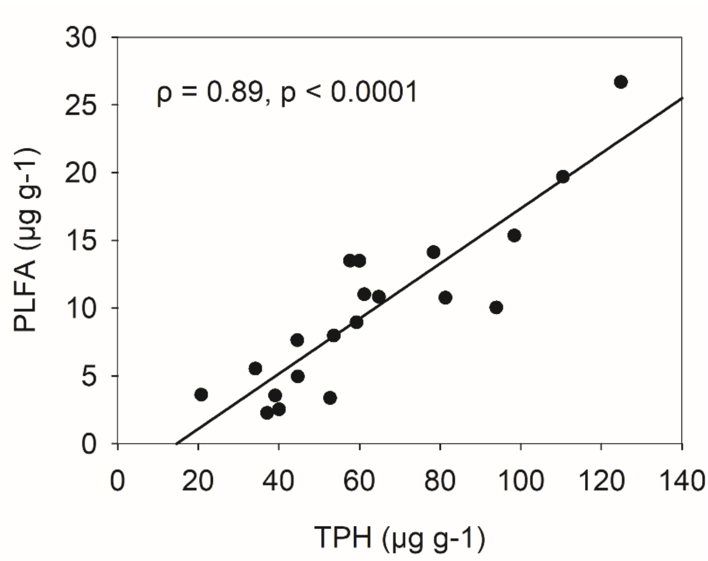

Figure S1: Scatter plot of microbial phospholipid fatty acids (PLFA) and total petroleum hydrocarbons (TPH) concentration. Linear regression curve and its coefficient of determination R^2 is presented.

Table S7: Results of ANOVA; test of between-subject effects and post hoc test Tukey HSD of the dependent variable $\delta^{13}C$ of CO_2 and the CO_2 concentration.

Tests of Between-Subjects Effects - $\delta^{13}C$ of CO_2					
Source	Type III Sum of Squares	df	Mean Square	F	Sig.
Corrected Model	99034.644a	50	1980.693	62.755	.000
Intercept	2140.794	1	2140.794	67.828	.000
Filter material	5041.310	3	1680.437	53.242	.000
Time	77899.264	22	3540.876	112.187	.000
Filter material * Time	2550.592	25	102.024	3.232	.000
Error	4544.951	144	31.562		
Total	106843.620	195			
Corrected Total	103579.595	194			

Tukey HSD - $\delta^{13}C$ of CO_2				
Filter material	N	Subset		
		1	2	3
Expanded clay	45	-4.9933		
Sand	44		-1.8636	
Sand & biochar	43		.5581	

Sand+	63			17.1508
Sig.		1.000	.157	1.000

Tests of Between-Subjects Effects - CO_2 concentration					
Source	Type III Sum of Squares	df	Mean Square	F	Sig.
Corrected Model	710.757a	50	14.215	4.514	.000
Intercept	1832.616	1	1832.616	581.919	.000
Filter material	35.948	3	11.983	3.805	.012
Time	546.218	22	24.828	7.884	.000
Filter material * Time	121.408	25	4.856	1.542	.061
Error	444.046	141	3.149		
Total	3272.233	192			
Corrected Total	1154.803	191			

Tukey HSD – CO_2 concentration			
treatment	N	Subset	
		1	2
Expanded clay	43	2.7372	
Sand+	63	3.2160	3.2160
Sand	43	3.4581	3.4581
Sand & biochar	43		3.9209
Sig.		.207	.225

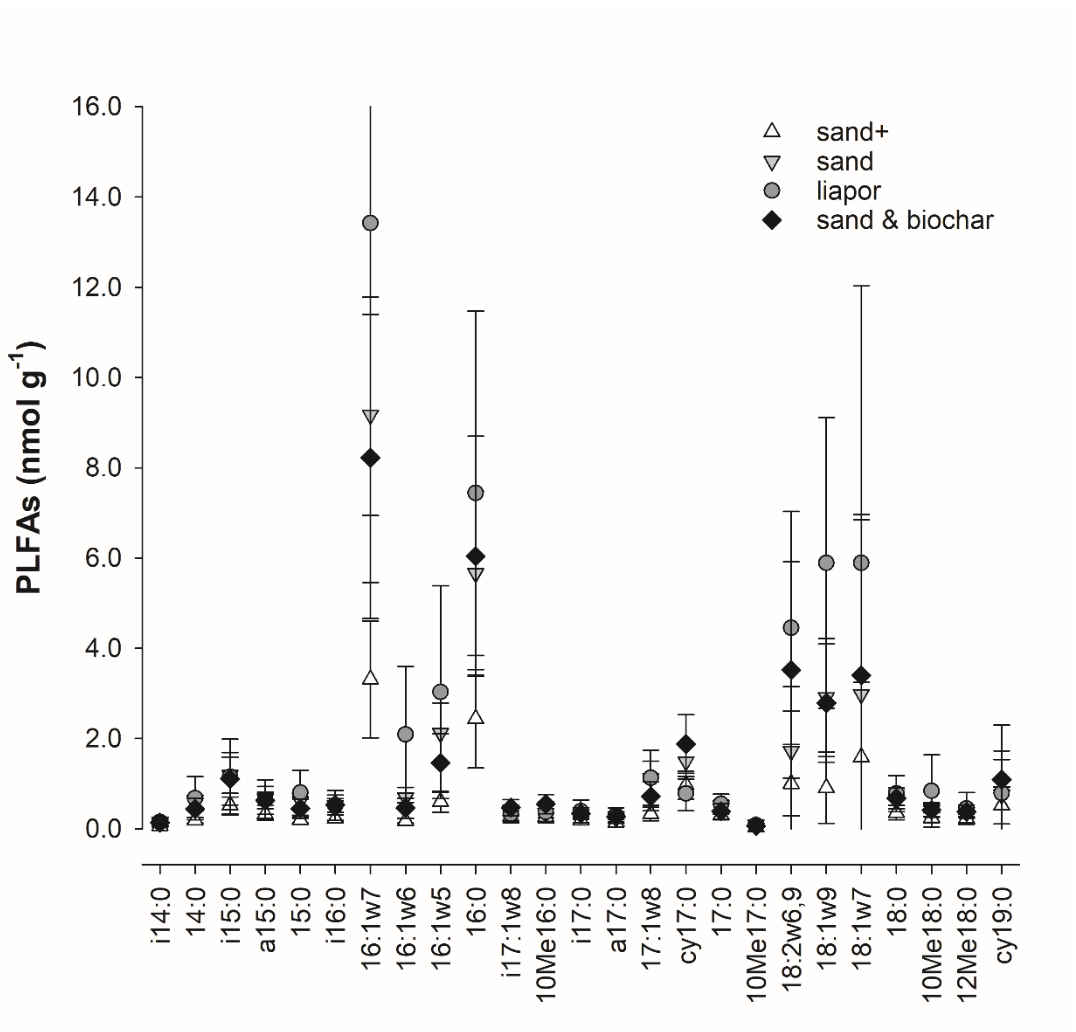

Figure S2: Concentration of the single microbial phospholipid fatty acids (PLFAs) in the different filters (0-10 cm) of the constructed wetlands. The error bars represent single standard deviation (n = 5).

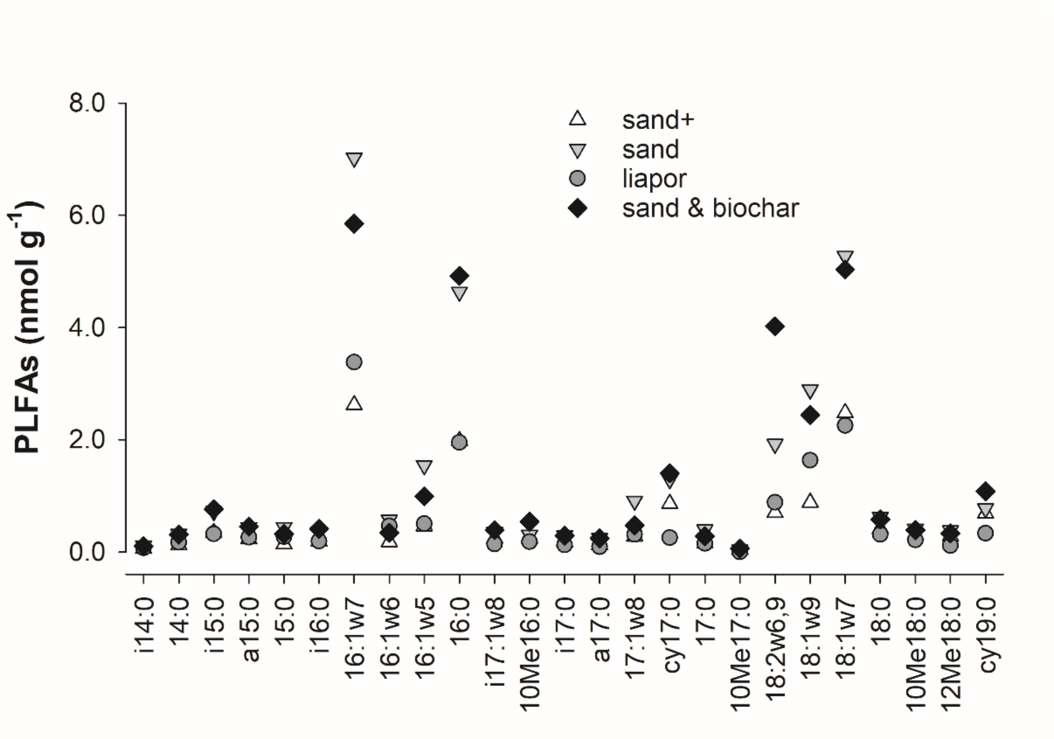

Figure S3: Initial concentration of the single microbial phospholipid fatty acids (PLFAs) of different filter materials in the microcosm experiment.

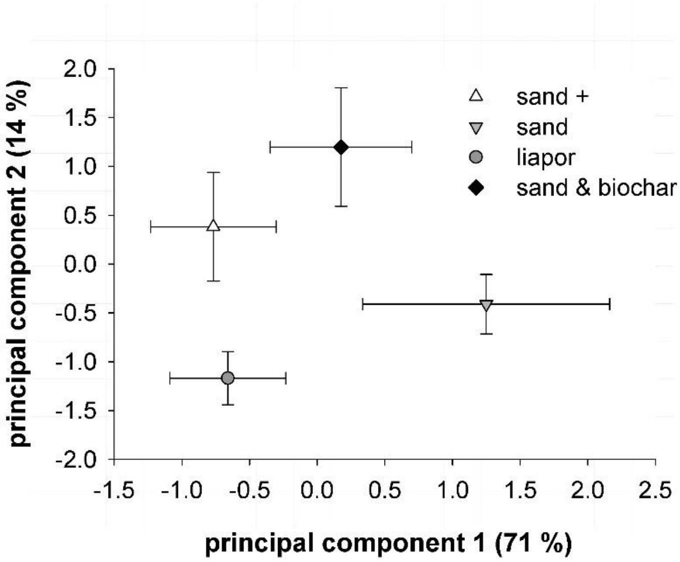

Figure S4: Principal component analysis of single microbial phospholipid fatty acids (PLFAs) of different filter materials in the microcosm experiment. The number in brackets give the percentage of total variance explained by the components.

Table S7: Bulk hydrogen stable isotope enrichment ε_{bulk}, enrichment at the reactive position ε_{rp}, AKIE and apparent biodegradation rates (k) were calculated using the day of headspace concentration decline as t_0 and fraction remaining (f = 1) and fitting a linear regression for three sand microcosm replicates (T = 12°C, 1 µl decane / g filter material, water-unsaturated). Slope of the regression ± standard error and the coefficient of determination (R^2) are reported.

Sand 3			Sand 4			Sand 5		
Days	f	δ^2H (‰)	Days	f	δ^2H (‰)	Days	f	δ^2H (‰)
1		-82	1		-82	1		-78
2		-83	2		-79	6		-77
5		-79	6		-79	14	1.00	-75
33		-81	14		-77	26	0.68	-79
40		-76	33	1.00	-75	33	0.58	-74
49	1.00	-76	40	1.00	-71	40	0.67	-74
55	0.95	-67	48	0.57	-70	49	0.37	-74
61	0.48	-61	55	0.57	-68	55	0.22	-73
62	0.17	-64	61	0.39	-64	61	0.16	-70
64	0.05		62	0.27	-52			
ε_{bulk}	-0.005 ± 0.005		ε_{bulk}	-0.016 ± 0.003		ε_{bulk}	-0.003 ± 0.001	
ε_{rp} [1]	-0.019 ± 0.017		ε_{rp}	-0.056 ± 0.012		ε_{rp}	-0.011 ± 0.002	
$AKIE_H$ [1]	1.129		$AKIE_H$	1.500		$AKIE_H$	1.072	
R^2	0.39		R^2	0.85		R^2	0.84	
k (day^{-1})	-0.067 ± 0.015		k (day^{-1})	-0.025 ± 0.004		k (day^{-1})	-0.017 ± 0.002	
R^2	0.87		R^2	0.92		R^2	0.93	

[1] n=22, x=6, z=6

Literature:

Elsner M, Zwank L, Hunkeler D, Schwarzenbach RP (2005) A new concept linking observable stable isotope fractionation to transformation pathways of organic pollutants. Environ Sci Technol 39:6896–6916. https://doi.org/10.1021/es0504587
